# Crossbridge Recruitment Capacity of Wild-Type and Hypertrophic Cardiomyopathy-Related Mutant Troponin-T Evaluated by X-ray Diffraction and Mechanical Study of Cardiac Skinned Fibers

**DOI:** 10.3390/ijms21103520

**Published:** 2020-05-15

**Authors:** Maki Yamaguchi, Masako Kimura, Tetsuo Ohno, Naoya Nakahara, Nobutake Akiyama, Shigeru Takemori, Naoto Yagi

**Affiliations:** 1 Department of Physiology, The Jikei University School of Medicine, Tokyo 105-8461, Japan; nkhr@jikei.ac.jp (N.N.); sml@jikei.ac.jp (S.T.); 2 Department of Integrated Physiology, Kagawa Nutrition University, Saitama 350-0288, Japan; masako@eiyo.ac.jp; 3 Department of Sports Medicine, Teikyo Heisei University, Chiba 290-0193, Japan; t.ohno@thu.ac.jp; 4 Core Research Facilities for Basic Science, The Jikei University School of Medicine, Tokyo 105-8461, Japan; nakiyama@jikei.ac.jp; 5 Spectroscopy and Imaging Division, Japan Synchrotron Radiation Research Institute, SPring-8, Hyogo 679-5198, Japan; yagi@spring8.or.jp

**Keywords:** cardiomyopathy, X-ray diffraction, troponin, skinned fiber

## Abstract

X-ray diffraction and tension measurement experiments were conducted on rat left ventricular skinned fibers with or without “troponin-T treatment,” which exchanges the endogenous troponin T/I/C complex with exogenous troponin-T. These experiments were performed to observe the structural changes in troponin-T within a fiber elicited by contractile crossbridge formation and investigate the abnormality of hypertrophic cardiomyopathy-related troponin-T mutants. The intensity of the troponin reflection at 1/38.5 nm^−1^ was decreased significantly by ATP addition after treatment with wild-type or mutant troponin-T, indicating that crossbridge formation affected the conformation of troponin-T. In experiments on cardiac fibers treated with the hypertrophic cardiomyopathy-related mutants E244D- and K247R-troponin-T, treatment with K247R-troponin-T did not recruit contracting actomyosin to a greater extent than wild-type-troponin-T, although a similar drop in the intensity of the troponin reflection occurred. Therefore, the conformational change in K247R-troponin-T was suggested to be unable to fully recruit actomyosin interaction, which may be the cause of cardiomyopathy.

## 1. Introduction

The regulatory mechanism underlying contraction by troponin-tropomyosin in striated muscle has not been completely elucidated yet. Deciphering the regulatory mechanism from a structural aspect is important for understanding the pathology of striated muscle diseases related to mutations of regulatory proteins, including familial cardiomyopathy. As for troponin, which is involved in the pathogenesis of familial cardiomyopathy, pieces of structural evidence that suggest formation of contractile crossbridges is involved in the regulatory mechanism of contraction have been accumulated to date [1,2,3,4,5,6]. However, the structural change of troponin in vertebrate striated muscle fibers is not well understood compared with that of tropomyosin [7,8,9]. Previous X-ray diffraction analyses on vertebrate skeletal muscle reported that the intensity of the meridional reflection of troponin at 1/38.5 nm^−1^ in the contracting state initially increases as a response to Ca^2+^ binding to troponin C (TnC) and then decreases as contractile crossbridges are formed [2,3,4,5,6]. However, in cardiac muscle, the change in intensity of the troponin reflection accompanied by crossbridge formation has not been clearly elucidated because obtaining a detailed X-ray diffraction pattern is far more difficult compared to skeletal muscle, due to the lower density of cardiac myofibrils and their susceptibility to a lack of oxygen.

**Figure 1:**
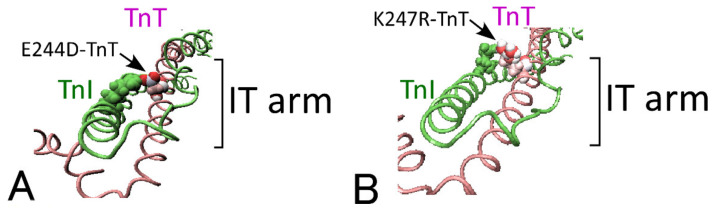
Atomic structure of cardiac troponin core domain visualized by VMD (version 1.9.3) based on the crystal structure (PDB ID; 1J1E). (**A**) troponin core domain with 244th amino acid (indicated by arrow) of TnT mutated from glutamate to aspartate (shown as space filling model in warm colors). (**B**) troponin core domain with 247th amino acid (indicated by arrow) of troponin-T (TnT) mutated from lysine to arginine (shown as space filling model in warm colors). TnT and troponin-I (TnI) in the “IT arm” domain are colored in pink and green, respectively. Residues in TnI that interacted with mutated residues in TnT are shown as space filling model in green.

In this study, we performed X-ray diffraction experiments on carefully prepared cardiac skinned fibers devised to obtain detailed X-ray diffraction patterns. Furthermore, we applied the method of “troponin-T (TnT) treatment” [10] to these cardiac skinned fibers, which exchange the endogenous troponin T/I/C complex with exogenously added TnT, to focus on the structural change of TnT, mutation of which is known to be an important cause of hypertrophic cardiomyopathy [11]. In TnT-treated fibers, we can detect structural change in troponin-T directly, as well as the crossbridge recruitment capacity independent of the function of Ca^2+^, since the inhibitory effect of troponin I (TnI), which can be released by Ca^2+^ binding to TnC under physiological conditions, is now removed by the exchange. First, we confirmed that the active actomyosin interaction caused by addition of ATP in TnT-treated fibers elicited a structural change in troponin-T in a cooperative manner, as has been reported with skeletal muscle [12,13]. Second, we evaluated the influence of the hypertrophic cardiomyopathy (HCM)-related mutations of TnT on the recruitment capacity of contractile crossbridges. We focused on the E244D-TnT and K247R-TnT mutants because both are autosomal dominant single amino acid mutations located in the “IT-arm” domain, which is the coiled-coil region of troponin (Figure 1), but they bring about different clinical symptoms: the patients who carry E244D-TnT exhibit mild hypertrophy [14] whereas those who carry K247R-TnT exhibit a complex phenotype of cardiomyopathy [15,16]. Equatorial X-ray intensities and tension measurement of TnT-treated cardiac fibers with the HCM-related mutants E244D-TnT and K247R-TnT revealed that K247R-TnT did not show any sign of the enhanced recruitment of actomyosin interaction that was shown with E244D-TnT, even though a similar amount of structural change in TnT occurred, implying that K247R-TnT induced abnormal contractility through disrupted linkage between the structural change of troponin and actomyosin recruitment.

## 2. Results

### 2.1. Intensity Change of Troponin Reflection of Cardiac Muscle Fibers with No Treatment 

**Figure 2:**
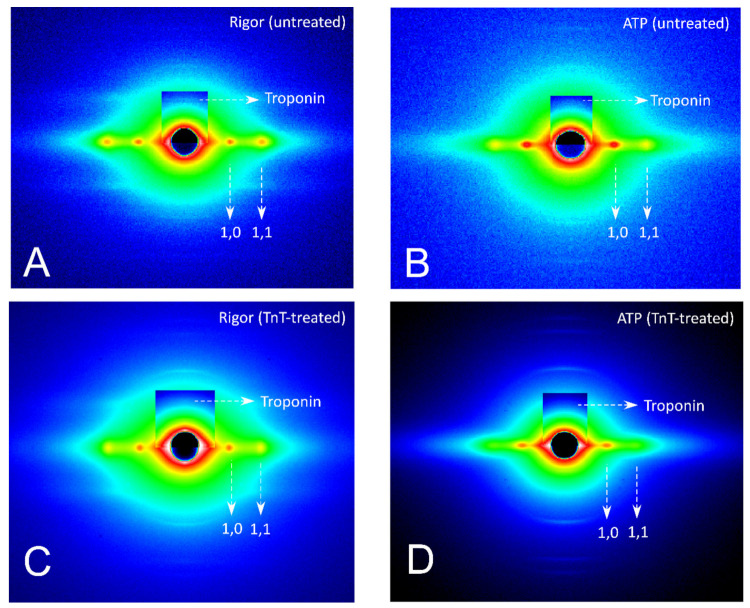
(**A**,**B**) Representative X-ray diffraction patterns obtained from rat cardiac skinned muscle fibers in the Rigor (**A**) and ATP (**B**) solutions without TnT-treatment. Meridional reflection of troponin at 1/38.5 nm^−1^ and equatorial reflections of 1.1 and 1.0 are indicated by arrows. **C**–**D**: Representative X-ray diffraction images obtained from rat cardiac skinned muscle fibers in the Rigor (**C**) and ATP (**D**) solutions after TnT-treatment with wild-type TnT. Meridional reflection of troponin at 1/38.5 nm^−1^ and equatorial reflections of 1.1 and 1.0 are indicated by arrows.

**Figure 3:**
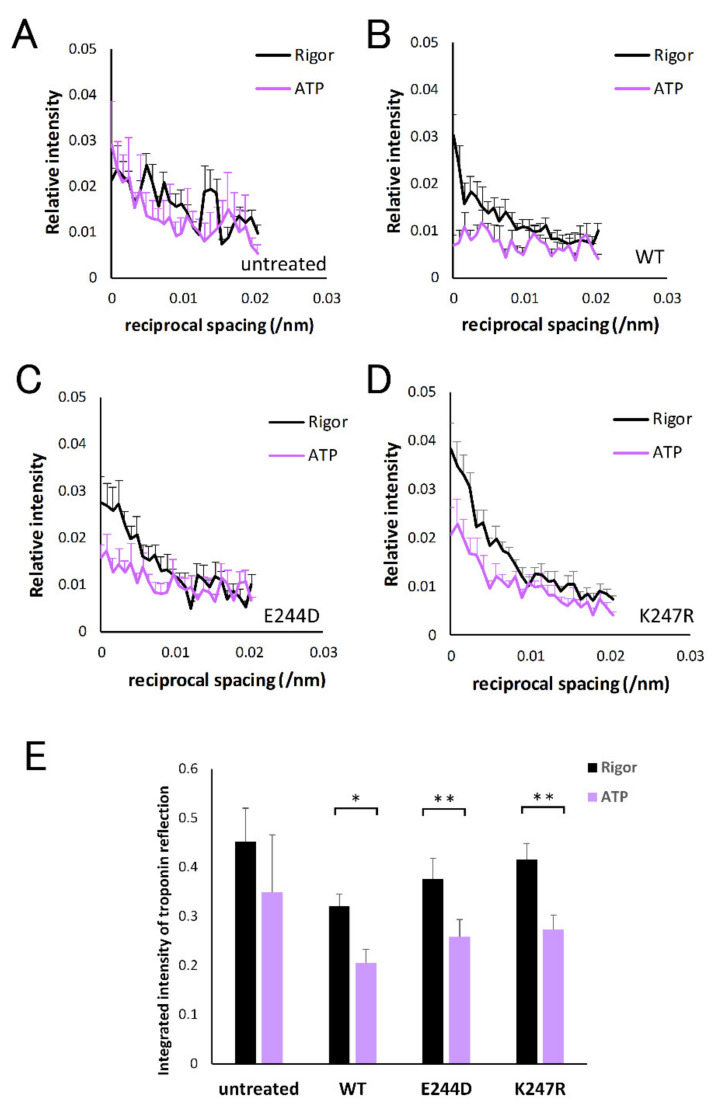
(**A**–**D**) Intensity profiles of the 38.5 nm troponin reflection of cardiac skinned muscle fibers along the equatorial axis in the Rigor (black) and ATP (purple) solution with or without TnT-treatment. Intensity is represented as relative value to integrated intensity of 5.9 nm actin layer line in the Rigor solution for each fiber. (**A**) Without treatment. Averaged values and standard error of the mean (S.E.M.) from five fibers are shown. (**B**) TnT-treatment with wild-type TnT. Averaged values and S.E.M. from eight fibers are shown. (**C**) TnT-treatment with E244D-TnT. Averaged values and S.E.M. from nine fibers are shown. (**D**) TnT-treatment with K247R-TnT. Averaged values and S.E.M. from nine fibers are shown. (**E**) Integrated intensities of the 38.5 nm troponin reflection of cardiac skinned muscle fibers in the Rigor (black) and ATP (purple) solution with or without TnT-treatment by wild-type TnT, E244D-TnT, or K247R-TnT. Averaged values and S.E.M. from eight (wild-type TnT treatment), nine (E244D-TnT treatment), and nine (K247R-TnT treatment) fibers are shown. * and ** indicate significant difference with *p* < 0.02 and *p* < 0.01 by paired *t*-test respectively.

Diffraction patterns of cardiac muscle fibers with no treatment before and after addition of ATP are shown in Figure 2A (before) and B (after). Before addition of ATP, the 1.1 reflection of cardiac muscle fiber was stronger than the 1.0 reflection, suggesting that myosin heads are bound to actin, forming “rigor crossbridges” owing to the lack of ATP. After the addition of ATP, the intensity of the 1.1 reflection decreased and that of the 1.0 reflection increased, indicating that rigor crossbridges were broken and the fibers relaxed. At 1/38.5 nm^−1^ on the meridional axis, troponin reflection was observed (horizontal arrow). In Figure 3, the intensity profiles of the troponin reflection along the equatorial axis (Figure 3A) and integrated intensity of the reflection before and after the addition of ATP (Figure 3E) are shown. Although the addition of ATP slightly affected the intensity profile, the change was not significant, suggesting that dissipation of rigor crossbridges by ATP addition did not significantly affect the conformation of troponin.

### 2.2. Intensity Change of Troponin Reflection of Cardiac Muscle Fibers after Treatment with Wild-Type TnT

Next, we examined the diffraction patterns from TnT-treated cardiac muscle fibers. Diffraction patterns obtained from cardiac fibers treated with wild-type TnT before and after the addition of ATP are shown in Figure 2C,D. Measurement of the intensity profile of the 38.5 nm troponin reflection (Figure 3B) and integrated intensity (Figure 3E) of fibers treated with wild-type TnT revealed that the intensity of the troponin reflection was significantly weakened by the addition of ATP. Although tension was not monitored in the X-ray experiments, considering the reported evidence that the muscle fibers after TnT-treatment start active contraction with the addition of ATP [17], the significant decrease of troponin intensity by ATP addition observed in the TnT-treated cardiac fibers can be attributed to formation of contractile crossbridges.

### 2.3. Intensity Change of Troponin Reflection of Cardiac Muscle Fibers after Treatment with The Mutant TnT

Finally, we examined the changes in the diffraction pattern of cardiac fibers treated with E244D-TnT and K247R-TnT mutants to obtain information about the pathological mechanism of HCM-related TnT mutations. The intensity profile of the troponin reflection along the equatorial axis and integrated intensity of TnT-treated cardiac fibers with E244D-TnT and K247R-TnT are shown in Figure 3C,D,E. As was the case with fibers treated with wild-type TnT, fibers treated with E244D-TnT and K247R-TnT showed reduced troponin intensity with ATP addition. Integrated intensity of the troponin reflection appeared slightly higher in the fibers treated with E244D-TnT and K247R-TnT than in those treated with wild-type TnT, both in the absence and presence of ATP, although the difference was not statistically significant.

### 2.4. Evaluation of Actomyosin Interaction of Cardiac Muscle Fibers with or without TnT-Treatment Based on Tension Development and Equatorial Reflection Measurement

**Figure 4:**
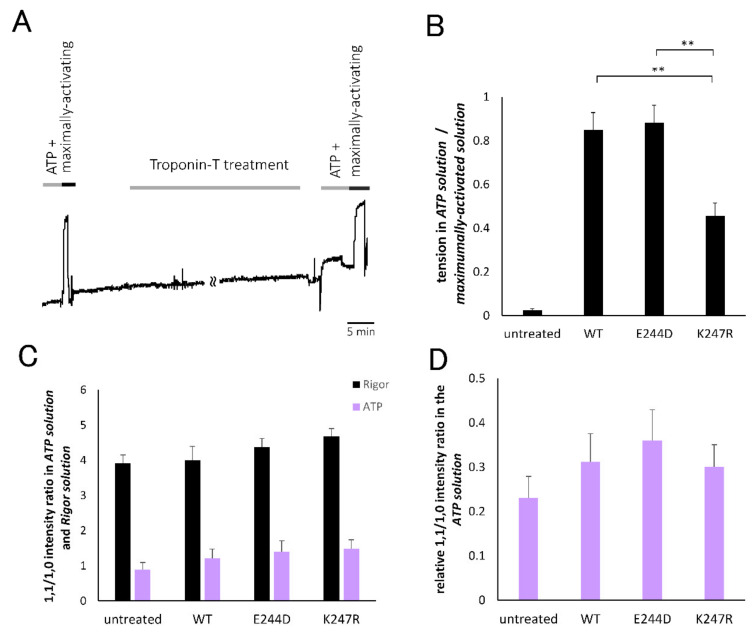
(**A**) Representative tension recording of cardiac skinned muscle fibers before and after TnT-treatment. The fibers in the ATP solution were transferred to Maximally-activating solution (during the period indicated as the black bar labeled “Maximally-activating”) to check contractility of the fibers, then TnT-treatment was conducted (during the period indicated as gray bar labeled “troponin-T treatment”). After the TnT-treatment, the fiber was transferred to Rigor solution, then soaked in ATP solution (during the period indicated as the gray bar labeled “ATP +”) to measure amplitude of Ca^2+^-independent tension. Finally, the fiber was soaked in Maximally-activating solution (during the period indicated as the black bar labeled “maximally-activating”) to measure amplitude of maximally developed tension of the fibers. (**B**) Summarized results of relative tension in the presence of ATP (ATP solution) to maximally developed tension in the presence of ATP and Ca^2+^ (Maximally-activating solution). Averaged value and S.E.M. for five (without treatment), seven (treatment with wild-type TnT), seven (treatment with E244D-TnT), and nine (treatment with K247R-TnT) fibers. ** represents *p* < 0.01 by ANOVA. (**C**) Intensity ratio of the 1.1 and 1.0 equatorial reflections of the cardiac skinned muscle fibers in Rigor (black) and ATP (purple) solutions before and after TnT-treatment with wild-type, E244D-, or K247R-TnT. Averaged values and S.E.M. from five (without treatment), eight (wild-type), nine (E244D-TnT treated group), and nine (K247R-TnT treated group) fibers are shown. (**D**) Intensity ratio of the 1.1 and 1.0 equatorial reflections of the cardiac skinned muscle fibers in the presence of ATP before and after TnT-treatment with wild-type, E244D-, or K247R mutant TnT, represented as a relative value to the ratio in the absence of ATP. Averaged values and S.E.M. from five (without treatment), eight (wild-type TnT treatment), nine (E244D-TnT treatment), and nine (K247R-TnT treatment) fibers are shown.

To evaluate the number of recruited crossbridges in the fibers after the TnT-treatment, the tension generating capacity and intensity ratio of the 1.1 and 1.0 equatorial reflections were analyzed. As seen in the representative tension trace after the TnT treatment in Figure 4A, the fibers developed significant tension triggered by the addition of ATP. The relative amplitudes of the tension elicited by ATP, standardized by the maximally developed tension in the presence of ATP and sufficient amount of calcium, are summarized in Figure 4B. It was observed that the fibers treated with wild-type and E244D-TnT elicited active tension comparable to the maximally developed tension, which was qualitatively consistent with a previous report by Harada and Potters [18]. However, fibers treated with K247R-TnT generated significantly lower tension than the fibers treated with wild-type or E244D-TnT. As for the intensity ratio of the 1.1 and 1.0 equatorial reflections (1.1/1.0 intensity ratio) obtained from the X-ray diffraction experiments, which is a structural index of the number of crossbridges, a decrease in the intensity ratio following ATP addition was observed in all fibers, reflecting the dissipation of rigor crossbridges which had strongly contributed to the 1.1 intensity. The intensity ratio after ATP addition showed different values among the groups, suggesting a difference in the number of recruited contractile crossbridges caused by ATP. Figure 4D shows the relative value of the 1.1/1.0 intensity ratio; that is, the 1.1/1.0 intensity ratio in the presence of ATP relative to that in the absence of ATP for each fiber. This value was considered to reflect the population of recruited contractile crossbridges because the variation in the absolute intensity ratios among the specimens due to different filament overlap within the sarcomere, which affects the 1.1/1.0 intensity ratio, can be compensated. It was found that the relative value of the 1.1/1.0 intensity ratio showed the same qualitative characteristics as that of the tension development property (Figure 4B). This result strongly suggested that the actomyosin interaction elicited in the fibers treated with K247R-TnT is less extensive than in those treated with the wild-type and E244D-TnT mutant.

## 3. Discussion

### 3.1. Intensity Drop of Troponin Reflection Triggered by Contractile Crossbridge Formation

Significant intensity change of troponin reflection was induced by addition of ATP in cardiac muscle fibers after TnT-treatment, indicating that troponin-T undergoes a structural change triggered by formation of contractile crossbridges in the cardiac skinned fibers. It is known that regulation by the tropomyosin-troponin system is influenced by formation of active crossbridges [12,13], implying conformational change of troponin by crossbridge formation. The intensity decrease of the 38.5 nm troponin reflection observed in the present study is qualitatively consistent with observations obtained in skeletal muscles [2,3,4,5,6].

As for the intensity drop, Matsuo et al. [2], from their observation in frog skeletal muscle during twitch, suggested that the decrease in the intensity of the 38.5 nm troponin reflection would be attributable to the detachment of part of the troponin molecule from tropomyosin after myosin binding. Tamura et al. [4], based on their time-resolved X-ray diffraction experiment with rabbit skinned skeletal muscle fibers, also described that the intensity decrease in the 38.5 nm troponin reflection, which concomitantly happened with contractile crossbridges formation, could be attributable to loosening or detachment of the movable part of troponin by myosin binding, but two other factors are also attributed; decrease in contrast due to myosin attachment to the thin filament between two neighboring troponin complexes and order-to-disorder transition of the thin filaments [19].

Regarding this study, the intensity change of the 38.5 nm troponin reflection observed with TnT-treated fibers was considered to be due to a structural change of TnT because the remaining endogenous T/I/C complex is unlikely to cause a large intensity drop (Figure 3A,E). However, the two factors related to thin filament structure proposed by Tamura et al. may also be considered to participate in the observed intensity change of troponin reflection.

### 3.2. Implication for the Pathogenesis of the K247R Mutant Cardiac Fibers

The relative 1.1/1.0 intensity ratio and tension measurement suggested that cardiac fibers treated with K247R-TnT exhibited a weaker property of contractile crossbridge recruitment, contrary to the anticipation based on reports by Matsumoto et al. [20] and Ford et al. [21] that the maximum ATPase activity of cardiac myofibrils with K247R-TnT was higher than that of myofibrils with the wild-type TnT. This can be discussed from the standpoint of atomic structure. Although both mutations are located at neighboring positions in the “IT arm” region and considered to have normal main-chain structures [22], replacement of lysine with bulky arginine in K247R-TnT may lead to conformational instability that affects interaction between TnT and tropomyosin/actin more severely compared to replacement of glutamate with aspartate in E244D-TnT (Figure 1). This difference may underlie the distinct characteristics of the K247R-TnT and may not induce a large conformational difference that changes intensity of the troponin meridional reflection. Matsuo et al. [23], based on their quasi-elastic neutron scattering experiment, suggested that the troponin T/I/C consisting of K247R-TnT had larger conformational space compared with wild-type. In addition, Yamaguchi et al. [22] reported from a molecular dynamics simulation study that K247R-TnT was less capable of interacting with TnI than E244D-TnT. Taking these reports into account, alteration of the amino acid in K247R-TnT may lead to structural instability that severely disturbs interaction with accompanied proteins or subunits. Further studies, including evaluation of the biochemical interaction between the TnT mutants and tropomyosin/actin as well as structural analysis such as the recent work on atomic structures of troponin-tropomyosin complex on the thin filament [24], will clarify the details of how the disruption of linkage between the structural change of troponin and actomyosin recruitment occurs.

Next arises the question of how this weaker property of contractile crossbridge recruitment causes K247R-TnT to induce cardiomyopathy. It is notable that K247R-TnT was considered as an HCM-causing mutant based on the first report of this mutation in HCM-patients by Garcia-Castro et al. [15]; however, Møller et al. [16] reported later that a patient carrying the K247R-TnT mutation exhibited a phenotype of dilated cardiomyopathy with no history of HCM. Turning our attention to experimental evidence, although Matsumoto et al. [20] and Ford et al. [21] reported that fibers reconstituted with K247R-TnT and wild-type troponin I/C exhibited higher maximum ATPase rates than those reconstituted with wild-type troponin T/I/C, there have been no reports of tension increase so far in the K247R-TnT mutant. We infer that K247R-TnT does not enhance tension development at the steady state but confers a higher ATPase cycling rate leading to various pathological phenotypes depending on the condition of the heart function.

### 3.3. Pathogenesis of the E244E Mutant Cardiac Fibers

The results of the 1.1/1.0 intensity ratio and tension development experiments imply that introduction of E244D-TnT would confer the trend to elicit larger crossbridge recruitment than the wild-type TnT (Figure 4B,D). The enhanced capacity for contractile crossbridge recruitment has been reported by Harada and Potters [18] based on the observation that the maximum tension after TnT-treatment was enhanced in the E244D-TnT treated fibers compared with those treated with wild-type TnT. Our results support their idea that cardiac hypertrophy of the E244D-carrying patients is caused by enhanced activation due to increased actomyosin recruitment from a structural aspect.

Matsuo et al. [25], based on their X-ray scattering experiments, reported that a reconstituted thin filament containing E244D-TnT undergoes structural change in an abnormal direction in the activated state compared with wild-type TnT. Their observation could be related to the increased actomyosin recruitment capacity of the E244D-TnT mutant suggested in this study.

## 4. Materials and Methods

### 4.1. Preparation of Skinned Cardiac Fiber from Rat Ventricle

Male rats (Wistar, Sankyo Lab. Industry, Tokyo, Japan, 250–280 g) were used with approval of the Institutional Animal Care and Use Committee of The Jikei University (#20-049, 31 Oct 2008). The rats were deeply anesthetized by intraperitoneal injection of pentobarbiturates (50 mg kg^−1^), following which their hearts were rapidly excised and blood was discharged by beating in warmed and oxygenated artificial extracellular solution. Next, cardiac muscle fiber bundles were dissected from the left ventricle wall very carefully. Cell membrane was disrupted by treatment of the fibers for more than 3 h with artificial intracellular solution containing 0.5% triton-X 100.

### 4.2. Replacement of Endogenous Troponin T/I/C Complex with Exogenous TnT

Endogenous troponin T/I/C complex was replaced with exogenous TnT by treating demembraned cardiac fibers with excess recombinant TnT solution (1 mg/mL of recombinant TnT, 0.25 M KCl at pH 6.5) for an average period of 5 h [10]. Recombinant wild-type TnT, as well as mutants that carry the HCM-related mutations E244D and K247R, were prepared using the pCold vector and an mRNA source of human TnT. The expression vector used was pET3-d and the expression host was BL21 (DE3). The exchange rate of endogenous troponin T/I/C complex to exogenous TnT was estimated to be 66%, 58%, and 67% for treatment with TnT of wild-type, E244D, and K247R, respectively, based on the amount of TnI detected by electrophoresis of the specimens [26]. Using the *t*-test, no statistical difference was found in the exchange rates among the TnT-treated groups.

### 4.3. X-ray Diffraction Experiments

X-ray diffraction experiments were performed at BL45XU in SPring8 (Hyogo, Japan) using a setup described elsewhere [27] with slight modifications. Diffraction patterns were obtained from a bundle of cardiac skinned muscle fibers, the diameter of which was less than 175 µm. Diffraction patterns were recorded in the Rigor solution, which contained no MgATP and was nominally free of Ca^2+^, with ionic strength of 0.2 at pH 7.0, and in the ATP solution, which contained 3.5 mM MgATP and was nominally free of Ca^2+^,with ionic strength of 0.2 at pH 7.0, using an imaging plate system (BAS 2500, Fuji Xerox, Tokyo, Japan). The X-ray wavelength was 0.09 nm and the beam size was 250 µm in width and 150 µm in height. The specimen-to-detector distance was 1.8 m. The sarcomere length of each fiber bundle was adjusted to 2.3 µm on average by laser diffraction. Experiments were carried out at 20 °C.

### 4.4. Measurement of Tension Development in Cardiac Muscle Skinned Fibers

Tension development in the cardiac muscle skinned fibers with or without TnT-treatment was measured using a tension transducer (Aurora scientific. series 400A, Aurora, ON, Canada). To evaluate maximally activated tension of the fibers, Maximally-activating solution, containing 3.5 mM MgATP and 10^−4.4^ M Ca^2+^, with ionic strength of 0.2 at pH 7.0, was used.

### 4.5. Analysis of Diffraction Patterns

Diffraction patterns were analyzed using MATLAB R2016B. Each X-ray diffraction pattern was rotated around the center and averaged quadrantally. The intensity profiles of the 38.5 nm troponin reflection along the meridional axis were obtained as below. Intensity in 1/54.8–1/32.9 nm^−1^ along the meridional axis was integrated within a slice of 0.00081 nm^−1^ width in the equatorial direction. Then, two Gaussian distributions along the meridional axis were fitted to the profile to obtain the troponin-derived reflection separately from that of the C-protein-derived reflection for each slice. This was repeated after shifting the slice by 0.00081 nm^−1^ in the equatorial direction.

The obtained intensity of the troponin reflection for each slice was plotted along the equatorial axis in the range of 0–1/47.4 nm^−1^.

The intensity profile of the 5.9 nm actin layer line was obtained as below. Intensity in 1/5.6–1/6.3 nm^−1^ along the meridional axis was integrated within a slice of 0.010 nm^−1^ width in the equatorial direction and the peak area above the background line between the bottoms at either side of the peak was obtained for each slice. This procedure was repeated after shifting the slice by 0.010 nm^−1^ in the equatorial direction. The obtained intensity for each slice was integrated along the equatorial axis in the range of 0–1/4.9 nm^−1^. Intensity of the 38.5 nm meridional reflection of troponin was expressed relative to the total integrated intensity of the 5.9 nm actin layer line.

Equatorial intensity profiles were obtained by integrating intensities in the range of 0–1/61.7 nm^−1^ in the meridional direction. Next, intensities of the 1.0 and 1.1 equatorial reflections were obtained by fitting two Gaussian distributions on an exponential background to the integrated profile.

## 5. Conclusions

Through direct observation of structural changes in TnT within the cardiac fibers, the K247R-TnT mutation was suggested to have a lower recruitment capacity of contractile crossbridges, even though a similar level of intensity drop in the troponin reflection occurred. Thus, the alteration of the single residue, K247R, affected function of the tropomyosin-troponin regulatory system without a large conformational change in TnT.

Finally, it should be noted that this study is limited to the function of TnT. Its interaction with troponin I/C, which affects actomyosin kinetics and Ca^2+^ sensitivity, is beyond the scope of this study. Future work on cardiac muscle fibers with mutant TnT and normal troponin I/C will further clarify the molecular pathology of TnT-related cardiomyopathy.

## Abbreviations

TnT	Troponin T
TnI	Troponin I
TnC	Troponin C
HCM	Hypertrophic cardiomyopathy
S.E.M.	Standard error of the mean
